# Thoracic tumor resection combined with SVC replacement using autologous pericardium

**DOI:** 10.1186/s12957-019-1769-3

**Published:** 2019-12-21

**Authors:** Sicong Jiang, Hao Hu, Changying Guo, Feng Jiang, Xi Liu, Lang Tang, Jianjun Tang, Xiaoliang Cheng

**Affiliations:** 1 Department of Thoracic Surgery, Medical College of Nanchang University, Nanchang, 330000 Jiangxi China; 2 Department of Thoracic Surgery, Jiangxi Province Tumor Hospital, No. 519 Beijing east Road, Nanchang, 330006 Jiangxi China; 3 Department of Oncology, The Fifth Affiliated Hospital of Sun Yat-sen University, No. 52 Mei hua east Road, Zhuhai, 519000 Guangdong China

**Keywords:** Superior vena cava, Autologous pericardium, Thoracic tumor, Lung cancer, Resection, Postoperative

## Abstract

**Background:**

Invasion of the superior vena cava (SVC) by thoracic tumors and occurrence of SVC syndrome are often encountered in clinical practice; but the prognosis in these cases is poor. Replacement of the SVC with autologous pericardial tissue is rarely performed. In this study, we sought to investigate the postoperative outcomes of this rare procedure.

**Methods:**

We performed a retrospective analysis of six patients who underwent SVC replacement using autologous pericardial tissue between October 2010 and November 2016. We collected data on the patients’ pathological features, operative characteristics, postoperative outcomes, and survival.

**Results:**

All six patients were male with an average age of 52 years (range, 18–62 years). Three of the patients had lung cancer, one had stage III thymoma, and two had germinoma. Four of the six patients had mild or moderate superior vena cava compression and no corresponding clinical symptoms. The other two patients had severe compression and obvious symptoms of SVC syndrome, with the typical swelling of the face, eyelids, and upper extremities. All six patients underwent complete tumor resection, with two of the lung cancer patients undergoing right lobectomy and one undergoing right pneumonectomy. With respect to the postoperative outcomes, one patient died, whereas the others did not develop any major complications. At the end of the follow-up period, five of the patients were alive and none of the patients had developed thrombosis in the grafts.

**Conclusions:**

Our findings indicated that SVC replacement with autologous pericardium is technically feasible and safe, with few postoperative complications and favorable long-term effects. Although it has some limitations, this method appears to be useful in achieving SVC reconstruction of moderate size. SVC replacement with autologous pericardium appears to have the potential for widespread clinical use.

## Introduction

Locally advanced lung or mediastinal tumors sometimes invade the superior vena cava (SVC), and surgery for such invasive lesions is still controversial because of the poor prognosis [1, 2]. Locally advanced lung or primary mediastinal malignancies are the most common causes of SVC syndrome, accounting for 60% of the cases [3]. However, improved surgical techniques and neoadjuvant therapy have made it possible to eliminate the clinical symptoms as well as achieve favorable immediate and long-term outcomes [2, 4].

Surgical treatment for the management of lung cancer or mediastinal malignancy invading the SVC involves the replacement of the SVC. The use of autologous tissue, particularly the pericardium, is less common and challenging in a clinical setting, especially when performed by surgeons who lack relevant experience [5]. Cases of SVC replacement in which the use of autologous pericardium is being considered need to be carefully evaluated for the applicability of the biological graft, survival status, and risk of complications after surgery. As early as 1973, Miller et al. [6] have shown that SVC replacement could be successfully performed in dogs by using an autologous pericardium. Subsequently, in 1990, Piccione et al. [7] reported the first successful SVC replacement using an autologous pericardium in humans.

Although successful SVC replacement with an autologous pericardium has been reported in a few cases, the application of this technique in actual practice still remains limited. In this study, we examine in detail the process of pericardial replacement of the SVC and summarize the advantages and long-term results of this procedure.

## Materials and methods

### Patient selection

**Fig. 1:**
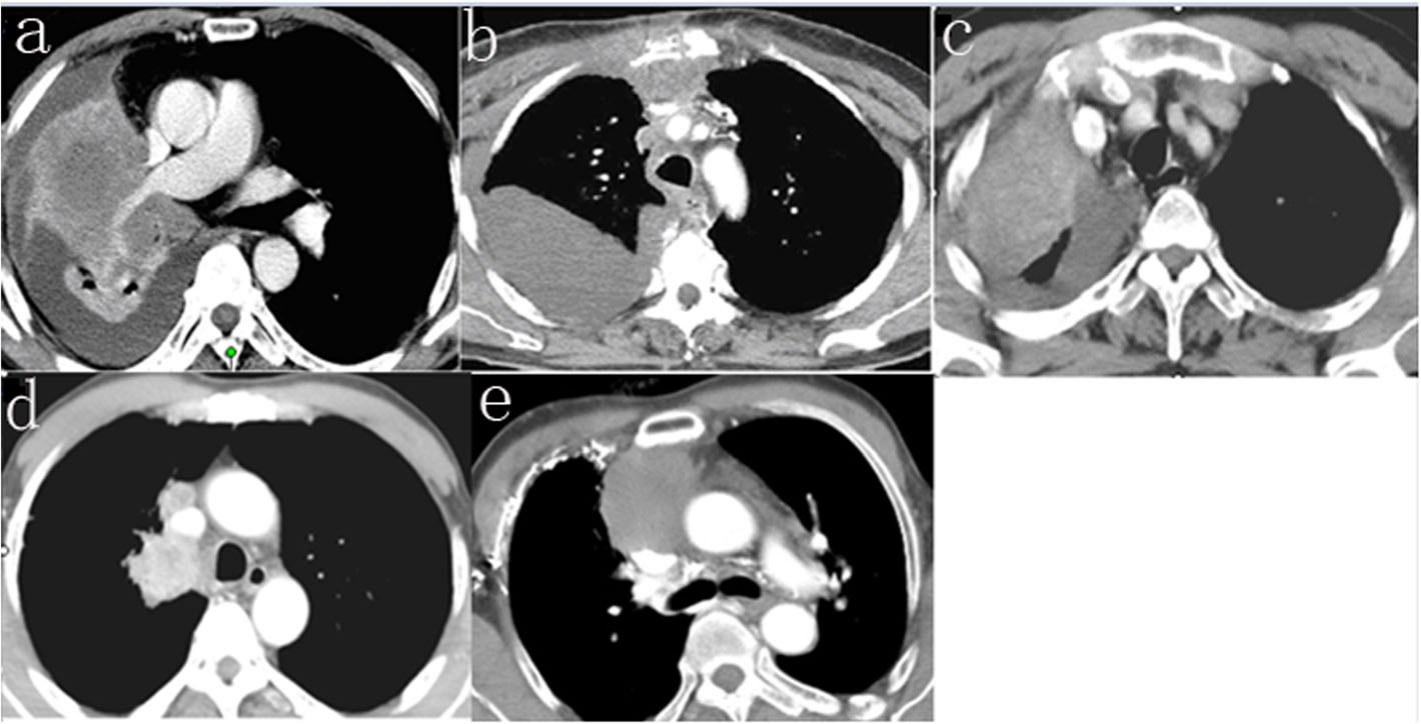
Tumors invading SVC on chest CT. **a** Right hilus pulmonis Sq.in patient 2. **b** Dysgerminoma in patient 3. **c** Right hilus pulmonis Sq.in patient 4. **d** Right upper lobe Sq.in patient 5. **e** Type AB thymoma in patient 6. *SVC* superior vena cava, *Sq.* squamous cell carcinoma, *CT* computed tomography

This investigation was designed as a retrospective study of the data of patients who underwent SVC replacement with an autologous pericardium at the Jiangxi Cancer Hospital, between October 2010 and November 2016. Three of these patients had lung cancer, one had thymoma, and two had germinomas. We analyzed the data of the patients to evaluate their clinical characteristics, pathological features, type of surgery, postoperative outcomes, and long-term survival rates. In addition, we assessed their imaging findings; representative images obtained from 2 of the 6 patients are presented in Fig. 1.

### Reconstruction process (two methods)

#### No invasion of the confluence in the left and right brachial veins

**Fig. 2:**
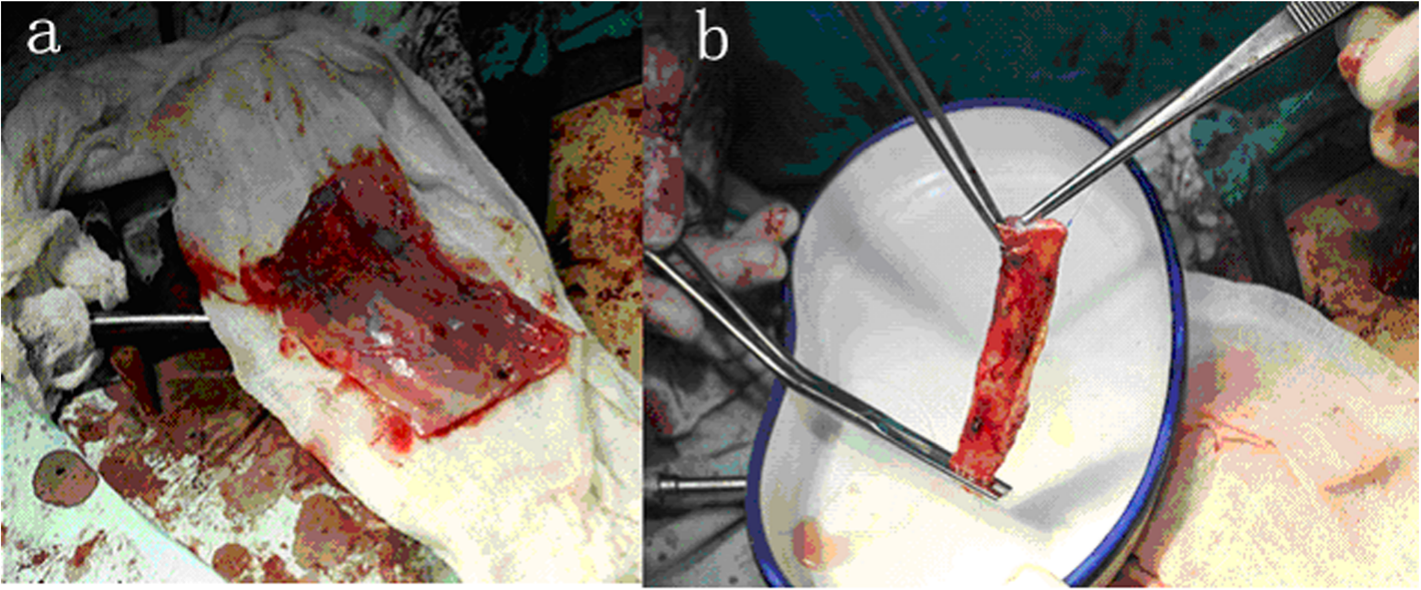
**a** Suitable size of autologous pericardium obtained and repeatedly washed with heparin saline. **b** The autogenous pericardium sutured to form a tubular pericardium

**Fig. 3:**
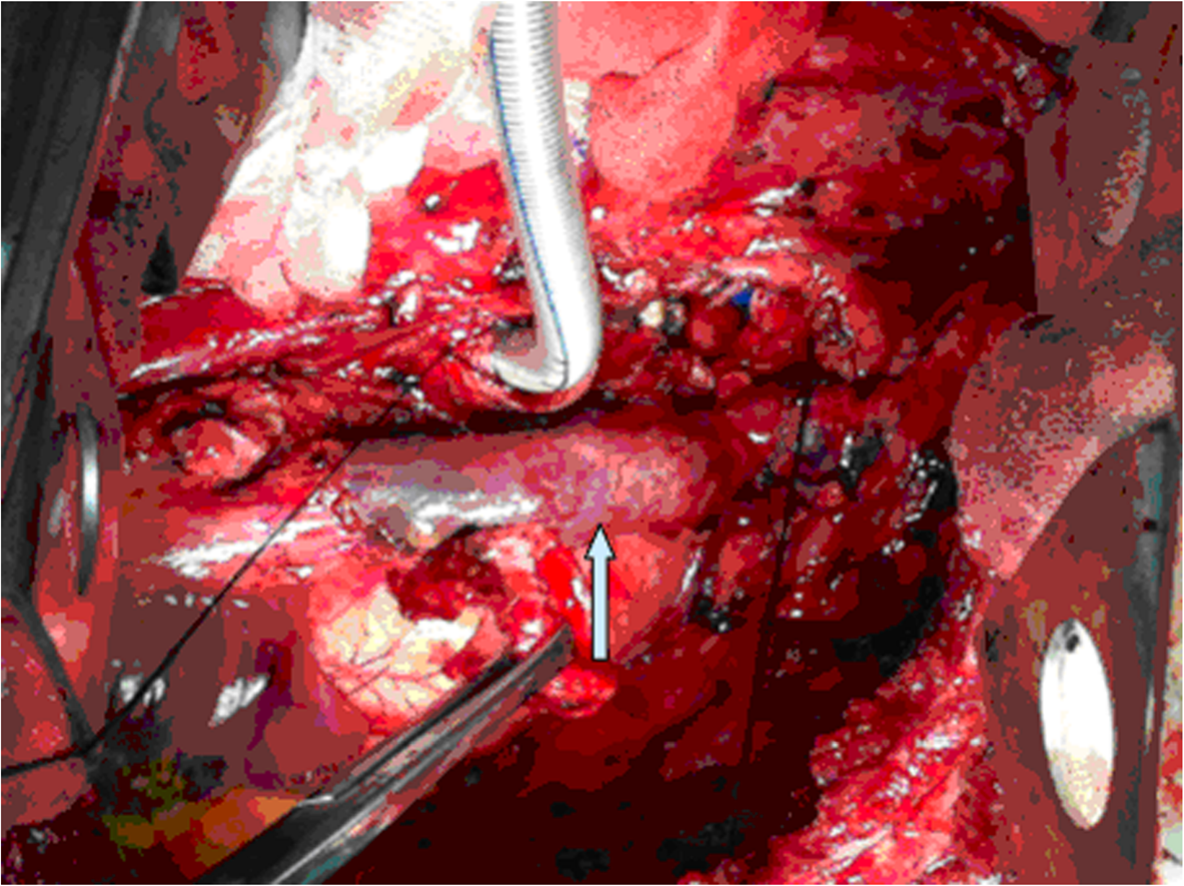
Reconstruction of the superior vena cava with a tube of autologous pericardial (*white arrow*)

**Fig. 4:**
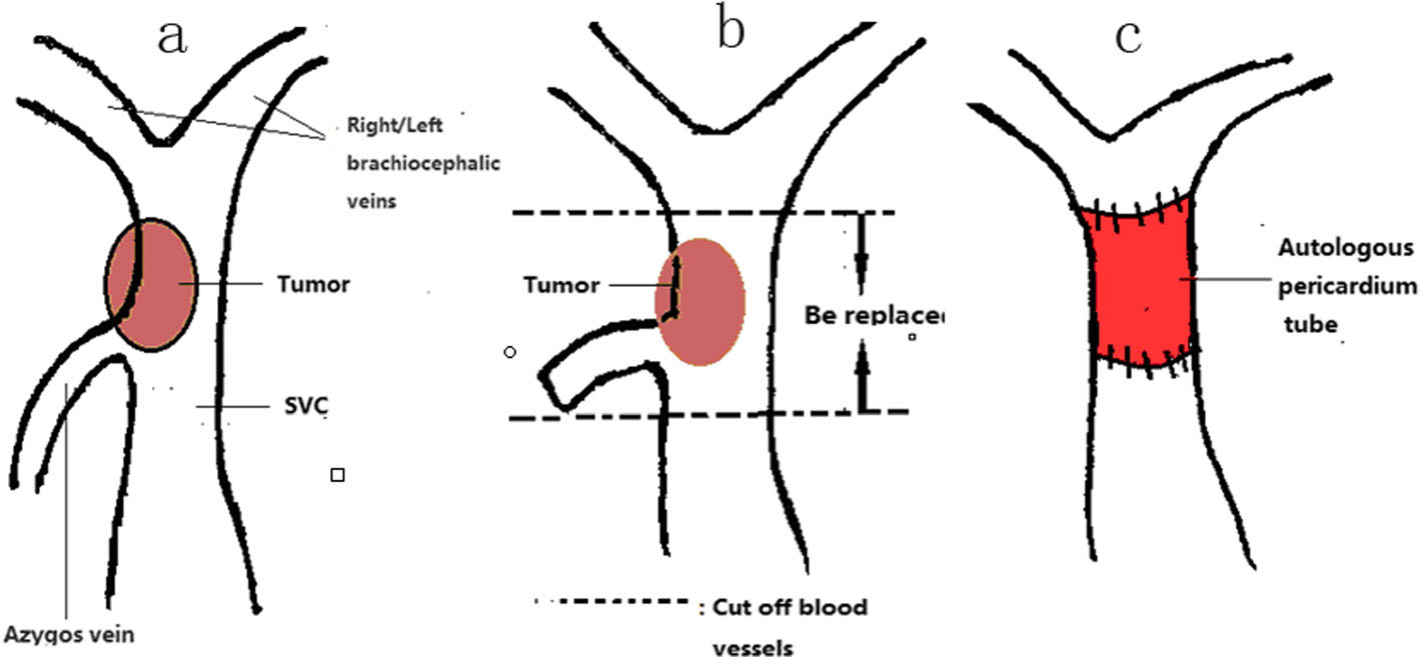
**a** Tumor invading the SVC, but not reaching the confluence of the right and left brachiocephalic veins. **b** SVC severed at the upper and lower ends of the tumor. **c** Autologous pericardial tube anastomosed to the distal and proximal ends of the SVC

In the patient who underwent SVC reconstruction for a thymoma, we performed a median sternotomy with an incision in the right first intercostal space. For the three lung cancer patients, a right posterolateral incision was taken on the fifth intercostal space. The arteries, veins, and bronchi of the lungs were prepared routinely, and the lung tissues were removed. The azygos vein was ligated and severed, with proximal and distal clamping of the SVC. We then resected the tumor-invaded portion of the SVC. Depending on the extent of the SVC resected, an appropriate length of the pericardium was obtained, from the precordial region. A 5-0 prolene suture line was used for the continuous eversion suturing of the two edges of the pericardium to form a tubular pericardium (Fig. 2). The inner and outer surfaces of the pericardial tube thus prepared were washed repeatedly with 1:1000 heparin saline. By placing additional continuous eversion sutures with the 5–0 prolene line, the two ends of the pericardial tube were anastomosed to the ligated distal and proximal ends of the SVC, taking care to eliminate any torsion. The lumen of the tube thus created was washed repeatedly with heparin saline, after checking for any kinks or trapped gas within (Fig. 3). The blockage of the SVC was removed, and filling of the pericardial tube was closely observed to rule out any bleeding through the anastomoses. The initial steps of reconstruction are shown in Fig. 4.

#### Invasion of the upper left and right brachial vein junctions

**Fig. 5:**
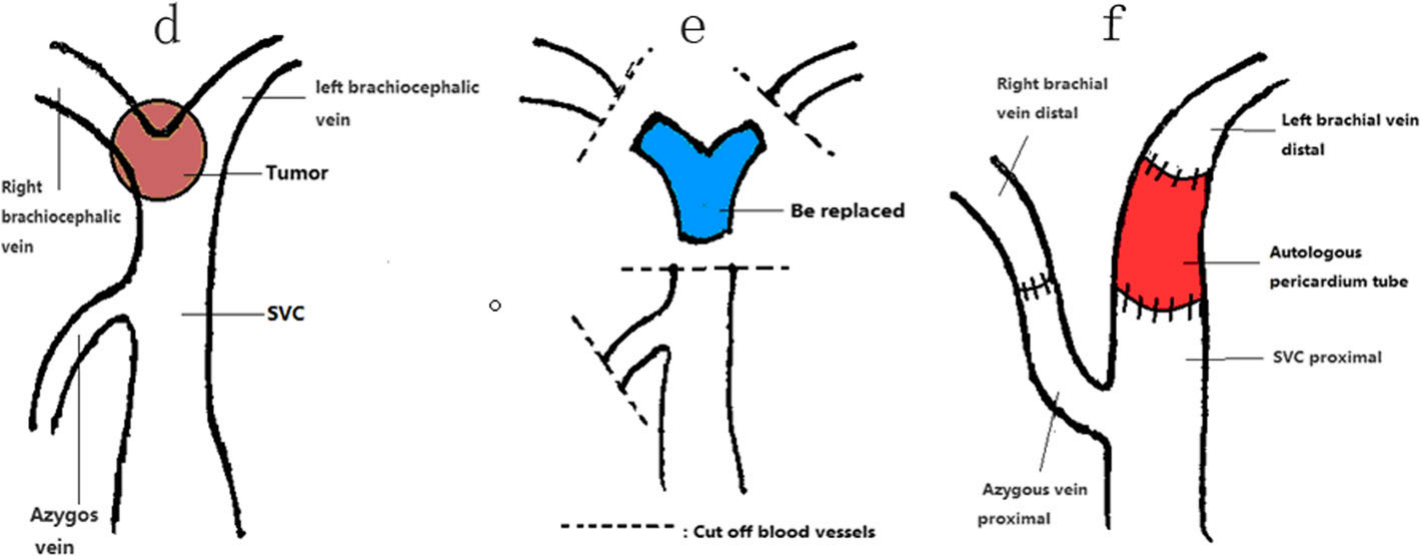
**d** The tumor invaded the SVC and reach the confluence of the right and left brachiocephalic veins. **e** Severed right and left brachiocephalic veins, distal end of the azygos vein, and the SVC at a distance of ≥ 2 cm from the base of the tumor. **f** Right brachiocephalic vein and azygos vein proximal anastomosis: The two orifices of the pericardial tube anastomosed proximally to the SVC and distally to the left brachial vein

Two of the enrolled patients underwent surgery for germinoma. In these patients, we performed median sternotomy in combination with an incision in the right first intercostal space. Further, the distal and proximal ends of the azygos vein and right brachiocephalic vein were clamped and then severed. The graft obtained was flushed with heparin saline, and proximal ligation of the right brachiocephalic veins and distal ligation of the azygos vein were performed. By using a 5-0 prolene suture line, the distal end of the right brachiocephalic vein was anastomosed with the proximal end of the azygous vein by serial sutures. Then, the two veins were declamped, and the blood flow in the anastomosis was checked to ensure that it was smooth and that there was no bleeding. A portion of the pericardium was resected from the precordial region, according to the required size, and the two edges of the pericardium were approximated to form a tubular pericardium by continuous eversion suture using a 5-0 prolene line. The inner and outer surfaces of the pericardial tube were repeatedly washed with 1:1000 heparin saline. The left brachiocephalic vein was blocked and cut-off, followed by flushing of the section with heparin saline. One end of the pericardial tube was anastomosed to the distal end of the left brachiocephalic vein using a 5-0 consecutive prolene suture line. The SVC was then clipped and severed at a distance of ≥ 2 cm from the base of the tumor. The other end of the pericardial tube was anastomosed to the proximal end of the SVC, taking care to avoid any torsion. Finally, the lumen of the prepared tube was repeatedly washed with heparin saline, after checking for any trapped gas and kinking. The blockage of the SVC was then released, and the pericardial tube filling was checked to ensure that there was no bleeding from the anastomosis. The procedure of this reconstruction process is shown in Fig. 5.

### Postoperative management

After the operation, patients were administered short-term intravenous anticoagulants (infusion of heparin 8000 U plus normal saline 500 mL/day). Routine thoracic spiral computed tomography (CT) and 3D reconstruction was performed for all patients, to assess the patency of the graft after surgery. Procedure-related mortality was defined as death occurring within 30 days of the surgery or within the first 30 days of the initial postoperative hospitalization period. Survival was defined as the time from surgery to death or the last follow-up.

## Results

### Clinical and pathological characteristics

**Fig. 6:**
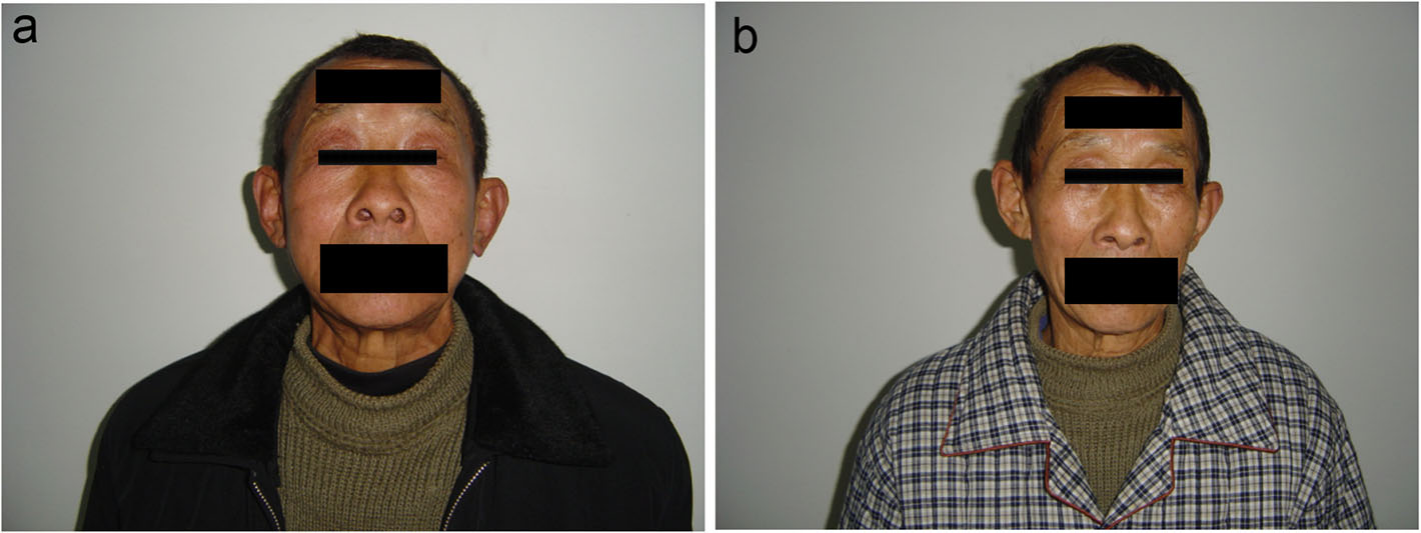
**a** Severe symptomatic superior vena cava (SVC) syndrome in a 57-year-old patient (#4). **b** Photograph of the patient 10 days after the SVC replacement with autologous pericardial; postoperative symptoms were significantly reduced. The clinical result is excellent 7 years after the operation

In our study, 2 of the 6 patients had developed manifestations of SVC syndrome before surgery, with the typical swelling of the face, eyelids, and upper extremities. After the surgery, symptoms had significantly reduced (Fig. 6). One of the enrolled patients underwent preoperative chemotherapy, whereas the others underwent postoperative chemotherapy and a stable disease (SD) was achieved in these patients, with no recurrence. At the time of presentation, the patients had varying degrees of SVC invasion of the tumors. The clinical and pathological characteristics of the six patients are shown in Table 1.

**Table 1:** Clinical and pathological characteristics of six patients

Pt no.	Age (years), sex	Tumor	Histology	Stage	TS (CM)	Treatment	SVC syndrome
1	18, M	Germ cell	Seminoma	/	12.7 × 9.5	S + CT + CT	No
2	56, M	Lung cancer	Sq.	T4N1M0 IIIA	10 × 7	S + CT + CT	No
3	57, M	Germ cell	Dysgerminoma	/	8 × 6	S + CT	Yes
4	53, M	Lung cancer	Sq.	T4N2M0 IIIB	12 × 22	S + CT + CT + CT	No
5	66, M	Lung cancer	Sq.	T4N1M0 IIIA	3.5 × 4.5	CT + S	No
6	62, M	Thymoma	AB thymoma	III	5 × 4	S + CT + CT + CT	Yes

*CT* chemotherapy, *M* male, *Pt No.* patient number, *S* surgery, *Sq.* squamous cell carcinoma, *TS* tumor size

### Surgical features

Intravenous sodium heparin (0.5 mg/kg) was administered before the clamping of the SVC. In five of the six cases, SVC reconstruction was performed using the SVC clamp technique; the average duration of SVC clamping in these cases was 77 min. In the remaining case, clamping was performed for 20 min combined with a venovenous shunt creation. The average operative time was 420 min, and the average volume of blood loss during the operation was 1050 mL. The amount of intraoperative blood transfusion required for the patients was different for different patients. Complete resection was performed for all patients with no instances of intraoperative death. The operative data for the six patients is shown in Table 2.

**Table 2:** Operative characteristics of six patients

Pt no.	Surgery	Approach	T (min)	CT (min)	OT (min)	IB (ml)	IBT (ml)
1	MTT	MS + RT		80	450	600	Autotransfusion (1000)
2	Right lobectomy	RPLT	/	70	400	1200	Erythrocyte (2 U) + adtevak (600)
3	MTT	MS + RT	/	75	420	2200	Erythrocyte (6 U) + adtevak (800)
4	Right lobectomy	RPLT	110	20	450	1000	Erythrocyte (2 U) + adtevak (400)
5	Right pneumonectomy	RPLT	/	90	375	500	/
6	ETT	MS + RT	/	70	430	800	Erythrocyte (2 U) + adtevak(600)

*CT* clamping time of superior vena cava, *ETT* extended resection of thymoma, *IB* intraoperative bleeding, *IBT* intraoperative blood transfusion, *MS* median sternotomy, *MTT* mediastinal tumor resection, *OT* operative time, *Pt No.* patient number, *RPLT* right posterolateral thoracotomy, *RT* right thoracotomy, *T* time to venovenous shunt, *U* unit

### Immediate and long-term outcomes after surgery

**Fig. 7:**
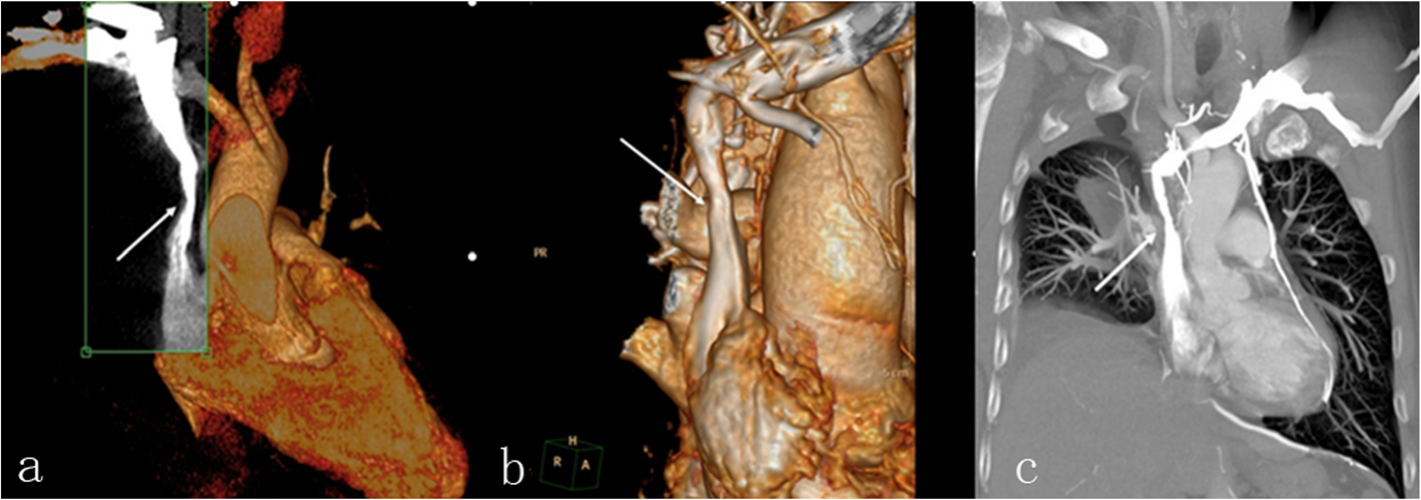
**a** and **b** Postoperative thoracic spiral computed tomography (CT) plus 3D reconstruction showing unobstructed, reconstructed SVC with adequate filling (*white arrow*). **c** CT venogram showing adequate postoperative filling of reconstructed SVC (*white arrow*)

The average length of hospital stay for five of our patients was 25 days. The average duration of postoperative anticoagulation was 10 days (range, 8–17 days). Postoperative thoracic spiral computed tomography (CT) with 3D reconstruction was performed in all cases, and uninterrupted flow and adequate filling of the SVC were noted in all cases (Fig. 7). The average follow-up duration was 55 months (range, 17–90 months), with only minor (non-life-threatening) postoperative complications and no serious complications. At the end of the follow-up period, five patients had survived. The sixth patient underwent right pneumonectomy and carinal reconstruction; on the first postoperative day, the patient developed respiratory failure and tracheotomy was performed immediately. However, it was ineffective and the patient was declared dead. Thus, the main cause of death in this patient was unrelated to the SVC reconstruction. The details of the immediate and long-term outcomes of surgery for all the six patients are provided in Table 3.

**Table 3:** Immediate and long-term outcomes after surgery in six patients

Pt no.	Complications	Mortality	Postoperative hospital stay (days)	Recurrence	Outcome	Graft patency	Survival (months)	PA (days)
1	None	None	12	No	Alive	Yes	17	8
2	None	None	20	No	Alive	Yes	90	10
3	None	None	21	No	Alive	Yes	87	8
4	None	None	32	No	Alive	Yes	23	8
5	Yes	Yes	/	/	/	/	1 day	/
6	None	None	40	No	Alive	Yes	59	17

*PA* postoperative anticoagulation, *Pt No.* patient number

## Discussion

For patients with malignant thoracic tumors that invade the SVC, treatment options such as chemotherapy, radiation therapy, resection of the SVC lesion, and bypass shunts [8, 9] have not proven to be sufficiently effective. Therefore, reconstruction of the SVC is the most important aspect of treatment in such cases. However, drawbacks of the surgical techniques and the high rate of rejection of transplant materials have resulted in postoperative mortality rates as high as 4.5–14% [2, 10, 11]. Nevertheless, some reports have been published on the successful reconstruction of the SVC with further improvements in surgical techniques and perioperative management [12–15].

Currently, the most common grafts used in SVC reconstruction are polytetrafluoroethylene (PTFE) Gore-Tex® synthetic prostheses and heterologous (bovine) custom-made pericardial prostheses [16–18]. Autologous pericardium grafts are currently rather unpopular, mainly because their preparation is difficult and time-consuming [19]. PTFE prosthesis offers the advantage of “rings,” which prevent graft collapse when the central venous pressure becomes negative [10]. However, it also has some disadvantages, mainly postoperative complications such as severe transplant rejection, early graft thrombosis, and high risk of infection. To prevent these complications, patients are required to take long-term anticoagulation and thrombolytic therapies.

With respect to the use of the bovine pericardial tissue, the main advantages are the lack of limitation in the length of the graft and lower risk of pericardial infection and thrombosis as compared to PTFE. However, Ciccone et al. [20] recommended the administration of continuous anticoagulant therapy for 6 months after surgery when bovine pericardium grafts are used. This is because bovine pericardium is heterogeneous and rejection at different levels of severity may still occur, which would increase the overall cost of the graft when compared with an autologous pericardium graft. Thus far, no reports have indicated the application of bovine pericardial tubes in SVC reconstruction [21]. Therefore, the safety and long-term effects of bovine pericardial tubes are still controversial.

One of the most important advantages of SVC reconstruction using an autologous pericardium is the material. Since this is an autologous tissue, the risk of antigenicity of other materials and rejection are completely eliminated. Furthermore, soon after implantation, the graft is re-epithelized with autogenous epithelial cells in humans, and it is associated with a low risk of infection, reduced platelet deposition, and less thrombogenicity on the flow surface. Thus, patients treated with this method do not require long-term, postoperative anticoagulant therapy.

The optimal duration for short-term anticoagulant therapy has not yet been established [22]. In our study, all 6 patients were empirically administered anticoagulant drugs for an average of 10 days, which greatly reduced the total duration of anticoagulation and significantly improved the patients’ quality of life. Because the length of the autologous pericardium is limited, it is suitable for SVC reconstruction when a moderate graft length is required.

In this paper, we describe, in detail, two surgical procedures for SVC replacement with an autologous pericardium. These procedures were useful in successfully creating unobstructed SVC grafts, without any serious postoperative complications. One of our patients was scheduled for right pneumonectomy and carinal reconstruction, which led to death during hospitalization. Spaggiari et al. suggest that pneumonectomy with complete resection of the SVC and reconstruction increases the risk of mortality in patients [11]. It must be emphasized that the mortality in our study group was due to pneumonectomy and oncological reasons and was not related to the SVC replacement with the autologous pericardium.

This study has a few limitations. The sample size is too small and larger-scale studies are necessary. Further, in the case of the pericardium, there is a limit to the size that can be utilized. This is a drawback of the replacement surgery. Nevertheless, the main purpose of this study was to propose an innovative surgical technique process, and this surgical technique has some obvious advantages and value.

## Conclusions

To conclude, we found that the use of other methods for SVC replacement could lead to serious postoperative complications, such as severe rejection. The choice of autologous pericardium would preclude some of these serious complications. We found that among the 6 patients included in this study, 5 had relatively few postoperative complications, which implies that there is still some predictability in the safety of the surgery. This study at least proves that it is feasible to use the autologous pericardium for the operation, and only interception of the pericardium makes it complex. There is a degree of predictability in terms of safety and efficacy with this method, and the replacement of the SVC with autologous pericardium seems to have broad clinical application potential.

## Data Availability

The datasets used and/or analyzed during the current study are available from the corresponding author on reasonable request.
